# Arterial shear rate determines the structure and mechanical properties of whole-blood clots

**DOI:** 10.1016/j.rpth.2026.106852

**Published:** 2026-07-14

**Authors:** Hande Eyisoylu, Parvin Ebrahimi, Marla Lavrijsen, Irène Nagle, Moniek P.M. de Maat, Gijsje H. Koenderink

**Affiliations:** 1 Department of Hematology, Cardiovascular Institute, Erasmus MC, University Medical Center Rotterdam, Rotterdam, Netherlands; 2 Department of Bionanoscience, Kavli Institute of Nanoscience, Delft University of Technology, Delft, Netherlands; 3 Department of Process and Energy, Faculty of Mechanical Engineering, Delft University of Technology, Netherlands; 4 Optical Image Centre OIC, Erasmus MC, University Medical Center Rotterdam, Rotterdam, Netherlands

**Keywords:** biomechanics, hemodynamics, microfluidics, thrombosis

## Abstract

**Background:**

Arterial thrombosis arises under high shear conditions and is a leading cause of ischemic stroke and myocardial infarction. The local hemodynamic environment during the formation of the thrombus influences its final composition, which in turn influences its mechanical characteristics and the associated risk of embolization. However, how arterial shear rates determine the local mechanical properties, structure, and composition of clots remains poorly understood.

**Objectives:**

This study investigated how physiological and pathologic arterial shear rates shape blood clot composition, formation dynamics, and regional mechanical properties in a microfluidic flow model.

**Methods:**

We used a microfluidic flow model that allows real-time imaging of blood clot formation under controlled arterial shear rates (300/s, 1000/s, and 3500/s). We visualized platelets and fibrin deposition using confocal microscopy and quantified composition-dependent spatial heterogeneities in clot stiffness with microindentation.

**Results:**

Pathologically high (3500/s) arterial shear rates promoted more rapid platelet aggregation and the formation of taller platelet-rich clots. Fibrin deposition started after ∼6 minutes in all shear conditions and coincided with platelet aggregate stabilization at higher shear rates. Microindentation revealed that platelet- and fibrin-rich regions were significantly stiffer than red blood cell–rich regions. Clots formed at 3500/s had a >3-fold higher average stiffness than those formed at 300/s, reflecting their higher platelet content.

**Conclusion:**

This study demonstrates that arterial shear rate governs clot growth and composition, translating into shear-dependent microscale mechanical properties. These findings provide new mechanistic insights into clot properties that are relevant for clot stability and vulnerability to embolization.

## Essentials

• Arterial blood clots form under flow, but effects on local clot strength are unclear.
• We formed human blood clots in a microfluidic model at arterial shear rates.
• We mapped local stiffness differences across clot regions with different composition.
• High shear formed taller, stiffer, platelet-rich clots, revealing flow-dependent clot mechanics.

## 1 Introduction

Arterial thrombosis is a critical underlying cause of cardiovascular events, such as myocardial infarction and ischemic stroke [1]. In arteries, thrombi arise under a high flow, often on ruptured atherosclerotic plaques that trigger thrombus formation via the exposure to thrombogenic materials such as tissue factor (TF), collagen, and von Willebrand factor (VWF) [1]. The local hemodynamic environment influences the initiation, propagation, and ultimate physical characteristics of the arterial thrombus. As blood flows through the arteries, variations in vessel diameter and flow velocity generate gradients in the vessel. Accordingly, within the arterial tree, shear rates can vary significantly, ranging from relatively low arterial shear (∼300/s in larger arteries) to moderate (∼1000/s in arterioles) and pathologic shear rates (>3000/s in stenosed vessels), while extremely high shear rates may reach beyond 10,000/s in severely stenotic cases [2,3]. This wide spectrum of arterial shear rates influences thrombus structure and composition, which will in turn influence mechanical properties of the thrombus [4].

Mechanical properties of arterial thrombi such as elastic modulus and fracture strength are major determinants of their mechanical stability, influencing the likelihood of embolization and the potential to successfully remove clots by mechanical thrombectomy [5]. Red blood cell (RBC)-rich thrombi require fewer number of trials to remove via thrombectomy compared with fibrin-rich thrombi [[6], [7], [8]]. Similarly, stroke thrombi that are rich in RBCs lyse more easily during intravenous thrombolysis [6,9]. On the contrary, fracture-mechanics studies have shown that fibrin-rich clot analogs have greater fracture toughness than RBC-rich clot analogs, suggesting a lower tendency to tear or fragment under mechanical loading [10]. Other studies have linked higher RBC content to clot fragility [11].

Blood composition, platelet reactivity, and arterial geometry, thus local shear and flow, vary between individuals. This complex interplay of different factors that influence clot properties obscures the specific contribution of flow to its composition, structure, and mechanics. This complexity motivates the use of *in vitro* models in which individual variables can be isolated and systematically tested under controlled conditions.

*In vitro* microfluidic models have become useful tools for studying thrombosis under flow conditions using small samples of human blood and providing controlled shear environments that approximate *in vivo* conditions. *In vitro* flow studies have shown that the shear rate determines the role of VWF in thrombus formation. At high arterial shear, VWF unfolds and binds platelet glycoprotein (GP)Ibα receptors to drive rapid tethering and aggregation, with subsequent stabilization via the integrin receptor GPα_IIb_β_3_ that binds fibrin [[12], [13], [14]]. At lower shear rates, thrombus formation is more coagulation driven, producing fibrin- and RBC-rich thrombi with fewer platelets [[15], [16], [17]]. Finally, several studies reported an increased occlusion capacity of thrombi with increasing shear rate [18,19].

However, a knowledge gap remains regarding how arterial shear rates influence the spatial heterogeneity of a thrombus’s mechanical properties. Most mechanical measurements of *in vitro* clots until now have been performed at a macroscopic scale [20]. With few exceptions [16,21], mechanical measurements have been restricted to clots formed under static conditions. These readouts are global, while stroke clots are known to exhibit marked spatial heterogeneity with distinct RBC-rich vs platelet-/fibrin-rich regions [22,23]. It is crucial to characterize the mechanical heterogeneities associated with this compositional heterogeneity for accurate predictions of *in vivo* embolization risk or thrombectomy outcomes. Therefore, it is necessary to link the dynamic processes of thrombus formation under arterial shear conditions to their effects on the local mechanical stiffness of the developing clot.

For this study, we used a microfluidic thrombus-on-a-chip model that allows precise control over the shear rate during clotting, mimicking physiologically relevant and pathologic flow conditions. Using this platform, we investigated thrombus formation under arterial shear rates spanning from physiological (300/s and 1000/s) to pathologic levels (3500/s), combining real-time confocal imaging of platelet and fibrin deposition with postclotting microindentation to map regional mechanical properties. By linking the arterial shear history of clot formation to the detailed composition, structure, and local mechanics, our work may help advance understanding and prediction of clinical outcomes such as clot stability and response to thrombectomy in arterial thrombosis.

## 2 Methods

### 2.1 Microfluidic model

#### 2.1.1 Device design

The microfluidic device used in this study was adapted from prior thrombosis-on-chip designs, including the larger-format geometry reported by Berry et al. [24] and was selected because it supports formation of tall occlusive clots while permitting postformation access for local mechanical testing. The 2 parallel channels have rectangular cross-sections with dimensions of 500 μm in width and 70 μm in height (Supplementary Figure 1), ensuring a height-to-width ratio that allows for platelet aggregation [25]. Additionally, the device contains a vacuum chamber that enables reversible bonding of the polydimethylsiloxane chip to the glass coverslip; upon release of the vacuum, the channel can be opened to directly access the formed clot for mechanical characterization. A detailed description of the device fabrication and assembly can be found in the Supplementary Information.

#### 2.1.2 Surface coating with collagen and TF

To simulate physiological thrombus formation, a thrombogenic spot was created on the surface of a glass coverslip (VWR; No. 1) by coating with collagen I and TF [26]. Briefly, a 2-μL droplet of Collagen I Horm suspension (Takeda; 0.1 mg mL) was pipetted onto the coverslip and left to dry for 1 hour in a humid environment at room temperature. Next, a 2-μL droplet of TF (Siemens Dade Innovin; diluted 1:30 in saline) was pipetted on the same spot and left to dry for another hour. To make sure the coated spot was consistent in size (1000 × 500 μm), a mask was taped over the glass coverslip. The coated spot was positioned centrally in 1 of the parallel arms of the device to direct clot formation to the desired region. Following coating, the device was incubated with bovine serum albumin (1%) to block any uncoated surface for 30 minutes.

### 2.2 Experimental setup

#### 2.2.1 Blood collection and preparation

Blood was collected via venipuncture from 4 healthy volunteers into 9-mL 3.2% sodium citrate tubes (Vacuette; Greiner Bio-one). There were 2 male and 2 female donors with an average age of 28 ± 1.5 years. Approval for the study was obtained from the local medical ethics committee (MEC-2021-0849). All experiments were conducted within 4 hours of the blood collection. Blood was labeled with DiOC_6_ (final concentration, 0.5 μg/mL; Anaspec) for platelets; with Alexa Fluor 647 fibrinogen (final concentration, 16.5 μg/mL; Molecular Probes; Life Technologies) for fibrin; and with fluorescently labeled (AF594) VWF antibody (Dako; 1:400). Immediately before blood was perfused through the channel, it was recalcified with CaCl_2_ (final concentration, 17 mM). Experiments using human blood were in accordance with established guidelines for thrombosis research [25,27,28].

#### 2.2.2 Realtime imaging of clot formation

Clots were formed under 3 different arterial initial shear rates (300/s, 1000/s, and 3500/s) controlled using a syringe pump (NE-1000 One Channel Programmable Syringe Pump). The wall shear rates, *γ* (per second), were computed from the volumetric flow rate *Q* (in milliliters per minute) using the Poiseuille solution for a rectangular duct [27] as follows:

(1) γ(persecond)=100Q/(h2w)

Shear rates of 300/s, 1000/s, and 3500/s refer to the initial shear rates calculated at the region of interest (ROI) where thrombus formation was quantified. These values were used to define the experimental flow conditions throughout the article. Due to the Y-shaped channel with 2 identical parallel arms, the volumetric flow rate was doubled in the syringe pump settings to achieve the desired wall shear rates. Blood was perfused through the channels and over the coated patch to initiate clotting. Flow was maintained for 10 minutes. To provide hemodynamic context for the flow conditions, we performed a supplementary computational fluid dynamics analysis comparing the channel at the start of perfusion (*t* = 0) and after clot growth (*t* = 10 minutes), with the clot represented as a luminal narrowing under each initial shear condition (Supplementary Figure 7).

Real-time imaging of clot formation was performed using a Leica TCS SP8 confocal microscope (Leica Microsystems) equipped with an HC PL APO CS2 20×/0.75 NA dry objective. To visualize platelet and fibrin deposition, 488- and 633-nm lasers were used, respectively. Image acquisition was conducted in a time-lapse *z*-stack mode with a scanning resolution of 512 × 512 pixels and a scan speed of 600 Hz.

To capture the entire height of the microchannel and the developing clot, *z*-stacks spanning 70 μm in depth were acquired at a step size of 2 μm. Time-lapse imaging was performed over a 10-minute period, with full *z*-stacks captured every 16 seconds. To ensure complete spatial coverage of the coated region, 2 ROIs were defined across the flow chamber based on the markings indicating the coated region. The confocal system was programmed to sequentially acquire *z*-stacks at ROI1 and ROI2 in alternating fashion throughout the imaging period, enabling near-continuous visualization of thrombus growth over time.

#### 2.2.3 Image analysis

All image processing and quantification were performed using Fiji (ImageJ; National Institutes of Health) [29]. Confocal *z*-stack time series acquired during clot formation were converted into maximum intensity projections for each time point (1-10 minutes). To isolate clot regions in the platelet and fibrin channels, automated thresholding was applied separately to each channel. After visual inspection of several thresholding methods (Supplementary Figure 2), the triangle algorithm was selected for the platelet channel, and the Otsu method was used for the fibrin channel, as these provided the most accurate segmentation of the clot structure in their respective channels. Following thresholding, we generated binary masks and quantified the percentage of the surface area covered by platelets and fibrin for each time point. From this analysis, we determined the kinetics of clot growth and the final clot composition. The platelet adhesion time was quantified as the time point where 2% of the surface was covered by platelets. This area coverage was selected since it clearly distinguished adhered platelets from flowing ones or noise. The fibrin network formation time was quantified from the %surface area coverage curves of the fibrin channel. The peak of the first-order derivative from this curve was selected as the time point to represent the network transition. This was also visually confirmed from the images as the point where fibrin(ogen) signal visually transitioned to a fibrous network structure. The clot height was quantified using the full *z*-stack at the final time point. The maximum height was defined as the vertical extent of continuous clot signal based on either channel. To assess the average lateral size of platelet aggregates, maximum intensity projections of the platelet channel at the final time point were used. Following thresholding and mask generation, we calculated the average size of individual platelet aggregates with the Analyze Particles tool in Fiji.

#### 2.2.4 Mechanical characterization

**Figure 1:**
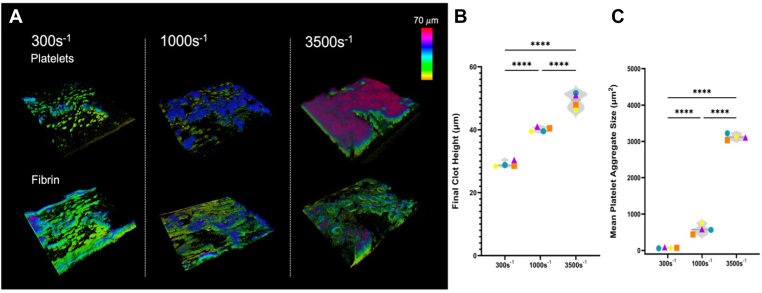
Final clot height and lateral platelet aggregate size vary with shear rate. (A) Representative 3D reconstruction of the final platelet aggregates (top row) and fibrin (bottom row) of clots formed under 300/s, 1000/s, and 3500/s. The 3D reconstructions are color coded for depth to better visualize clot height. Note that the fibrin network covers the platelet aggregates. (B) Clot height at varying shear rates, measured from the confocal *z*-stacks at the final (10-minute) time point. At higher shear rates, clots grow taller in height (each symbol represents a donor, averaged from minimum 4 replicates per donor). (C) Quantification of the lateral size of the platelet aggregates (from the platelet channel only) formed under varying arterial shear from maximum intensity projections (each symbol represents a donor, averaged from minimum 4 replicates per donor). Significantly larger platelet aggregates form at higher shear rates, especially for the highest shear rate (3500/s). ∗∗∗∗*P* < .0001.

After the clots were formed under either the lowest (300/s) or the highest (3500/s) shear rate, the vacuum bond was released, and the polydimethylsiloxane channel was carefully separated from the glass coverslip. The coverslips with the thrombi were submerged in HEPES (4-[2-hydroxyethyl]-1-piperazineethanesulfonic acid) buffer (10 mM HEPES, 115 mM NaCl, pH 7.4) to preserve the clot structure. To assess the local mechanical properties of the thrombi, microindentation was performed using a Chiaro Nanoindenter (Optics11) operated by the Piuma software (Optics11 Life). The microindentation experiments were conducted 2 to 3 hours after clot formation, allowing enough time for clot contraction. The indenter was mounted on an epifluorescence microscope (Leica Thunder) equipped with a 10× dry objective (HC PL APO 0.45NA; Leica) and monochrome sCMOS camera (Leica). The combined setup enabled us to correlate local indentation measurements with the local clot composition directly at the site of indentation. Clots were fluorescently labeled for platelets and fibrin (same as for confocal imaging) and imaged using a 200-mW solid-state LED5 light source (Leica), while RBCs were visualized via the brightfield channel. This visual guidance allowed us to classify indentation sites as fibrin- and platelet-rich, RBC- and fibrin-rich, or RBC-rich regions. Specifically, for each indentation region, we checked each channel (platelet [fluorescence], fibrin [fluorescence], and RBCs [brightfield]) for a signal. If there was a signal from any of these channels, the region was classified as rich in that component (for examples, see Figure 1D).

Indentations were performed in indentation control mode using a spherical probe with a radius of 56 μm and stiffness of 0.22 N/m (Optics11). Indentation mode was used to impose a predefined indentation (depth–time) protocol, during which the instrument continuously measured the corresponding load–displacement curve. Each indentation was performed at a loading and unloading rate of 2 μm/s, to an indentation depth of 2 μm, making sure that the maximum indentation depth was <5% to 10% of the sample thickness to avoid any influence of the substrate on the stiffness measurements.

The resulting force (*F*)–indentation (*δ*) curves were analyzed using the Hertzian contact model to calculate the effective Young modulus (*E*_eff_). Specifically, the loading branch was fitted to the Hertz model for a rigid sphere, indenting an elastic half-space [30] as follows:

(2) $$F=\frac{4}{3}E_{\text{eff}}R^{1/2}\delta^{3/2}$$

The effective Young modulus is related to the actual Young modulus *E* according to:

(3) $$E_{\text{eff}}=E/\left(1-\upsilon^{2}\right)$$

We assumed a Poisson ratio (*υ*) of 0.5, corresponding to an incompressible material. Curve fits were performed with the Optics11 Data Viewer software, which implements a least-squares fit of the measured *F* – *δ* data to Equation 2 (see Supplementary Figure 3 for example). Because clots are viscoelastic and heterogeneous, we interpreted *E*_eff_ as a comparative local stiffness rather than an absolute elastic modulus.

### 2.4 Statistical analysis

All image-based quantifications were performed using clots generated from 4 healthy donors, with 4 clots analyzed per donor for each initial shear condition (300/s, 1000/s, and 3500/s; *n* = 12 per donor and *n* = 48 clots in total). For each donor, 2 repeats per shear condition were performed on 1 day in an ascending shear order (300/s 1000/s, and 3500/s), and two additional repeats per shear condition were performed on the following day in a descending order (3500/s 1000/s, and 300/s) to minimize potential order bias. For mechanical characterization, microindentation testing was performed on a total of 9 clots (3 clots per 3 donors) for both the 300/s and 3500/s conditions, with at least 5 indentations conducted per clot in compositionally distinct regions identified by confocal imaging. Data are presented as mean ± SE. Statistical comparisons between shear conditions were performed using 1-way analysis of variance with Tukey post hoc test for multiple comparisons. For nonparametric data, the Kruskal–Wallis test with Dunn correction was applied. A *P* value of <.05 was considered statistically significant. All statistical analyses were conducted using GraphPad Prism (version 10; GraphPad Software).

## 3 Results

### 3.1 Real-time observation of clot formation

**Figure 2:**
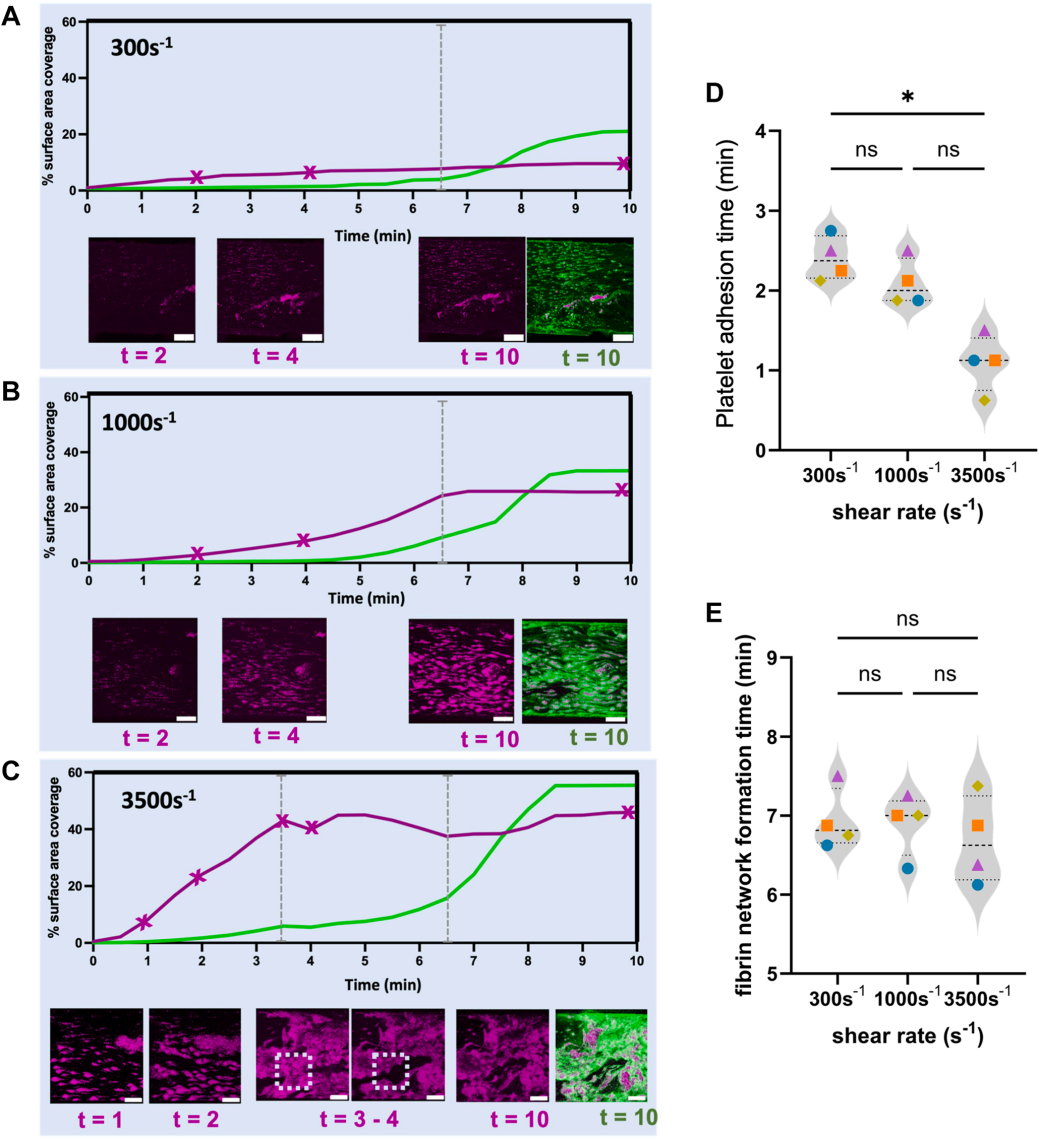
Temporal dynamics of platelet deposition and fibrin network formation under arterial shear conditions. Representative examples of the evolution of the fibrin (green) and platelet (magenta) surface coverage (in %) over a period of 10 minutes at shear rates of (A) 300/s, (B) 1000/s, and (C) 3500/s. Underneath the plots, maximum intensity confocal projections of platelets at early, mid, and late time points are shown (the corresponding time points [minutes] are indicated by the *x* signs on the magenta curve for each image) along with a merged maximum intensity projection of the final clot. The mid (3–4 minutes) time point is shown only for the 3500/s, to highlight the unstable platelet aggregation during this period. For the lower 300/s and 1000/s shear rates the early and the late time points are shown. (A) At 300/s, platelet adhesion begins within the first 2 minutes, forming small, discrete aggregates. Platelet surface coverage remains low throughout the 10-minute period. Fibrin network formation begins around 6 minutes (indicated by the dashed line). (B) At 1000/s, platelet surface coverage gradually increases, reaching a plateau after 6 minutes, coinciding with the onset of fibrin network formation (indicated by the dashed line). (C) At 3500/s, the platelets show rapid aggregation within the first 3 minutes, followed by a transient unstable phase (between the 2 dashed lines) that ends after 6 minutes, as the fibrin network starts to form and stabilize the clot. The confocal images show that during the unstable phase, platelets that were attached in the earlier time frame (shown inside the dashed square) are washed away in the next frame. Scale bars: 100 μm. (D) The platelet adhesion time was quantified as the onset of initial platelet adhesion (2% of the surface area coverage by platelets). Values for each flow rate are calculated by averaging minimum 4 replicate experiments from 4 individual donors. Platelet adhesion was significantly faster at 3500/s compared to both 300/s and 1000/s. (E) Fibrin network formation time was quantified as the time point for transition from fibrin(ogen) signal to a visible fibrous network structure. This time point was quantified as the point corresponding to the peak of the first-order derivative from the %area coverage curve. Fibrin network formation time showed no significant difference across shear rates and were between 6- to 8-minute marks.

We visualized clot formation in real time with confocal microscopy under conditions of arterial flow using a microfluidic flow model. Blood was flown into the device at different flow rates to achieve shear rates representing low (300/s) and mid (1000/s) normal arterial flow rates and a pathologically high flow rate (3500/s). Figure 2 shows representative confocal images of platelets and fibrin over time, comparing the clotting dynamics at the three shear rates. To quantify the temporal progression of platelet accumulation and fibrin deposition, we determined the percentage of surface area covered by platelets and fibrin, illustrated with representative examples underneath the curves. Representative movies showing 3-dimensional (3D) renderings of platelet, fibrin and merged channels over time for each shear rate are presented in Supplementary Movie 1.

At the lowest shear rate (300/s) (Figure 2A), platelet adhesion to the bottom of the microfluidic channel began within the first 2 minutes, forming small, discrete aggregates. Platelet surface coverage remained low throughout the 10-minute observation period. The platelet aggregates themselves also remained unchanged, showing no evidence of rearrangement or fragmentation. Fibrin deposition started after 6 minutes, as indicated by the dashed line (Figure 2A), typically around the platelet aggregates (Supplementary Figure 4). At the shear rate of 1000/s, platelet aggregates formed across the entire coated surface and were significantly larger than those observed at 300/s (Figure 2B). The platelet coverage gradually increased, reaching a plateau after 6 minutes. Fibrin deposition again began after 6 minutes, localizing around platelet clusters. At the highest shear rate of 3500/s, platelet adhesion and aggregation was much more rapid, with coverage rising sharply to ∼40% within 3 minutes (Figure 2C). However, following the rapid aggregation phase, platelet aggregates were unstable, showing flow-induced rearrangement and partial detachment (Figure 2C). This unstable behavior persisted until ∼6 minutes, after which the coverage of platelets stabilized. This stabilization moment coincided with the onset of fibrin deposition, which again started around 6 minutes. This unstable platelet adhesion phase was observed in 6 of 16 total clots at 3500/s and was not observed in any of the lower shear rate clots. Additionally, this unstable platelet aggregation was observed prior to a visible fibrin network formation. Fibrin deposition originated at platelet aggregates and gradually extended to interconnect the aggregates. The average platelet adhesion time per donor across shear rates in Figure 2D shows a significanty lower platelet adhesion time at 3500/s, whereas fibrin network formation time does not appear to vary with shear rate (Figure 2E).

Additionally, using the brightfield channel, we visualized RBC dynamics relative to platelet and fibrin architecture in real time. Supplementary Movies 4 to 6 of the brighfield channel show RBC entrapment over time. Following initial platelet adhesion and aggregation, RBCs began to colocalize with the platelet aggregates. As a fibrin network developed around these platelet aggregates and some RBCs, RBC retention became more stable, while free-floating RBCs were still able to flow around. For all shear rates, an initial platelet adhesion and aggregation over the coated surface was followed by RBC entrapment via a final fibrin network formation.

In summary, larger shear rates promote faster and more extensive platelet aggregation than lower shear rates. Across all shear rates, fibrin formation started after 6 minutes and was spatially organized around platelet aggregates, which acted as nucleation points for fibrin fiber extension and clot stabilization (Supplementary Figure 4).

### 3.2 Shear-dependent platelet recruitment is potentially mediated by VWF

**Figure 3:**
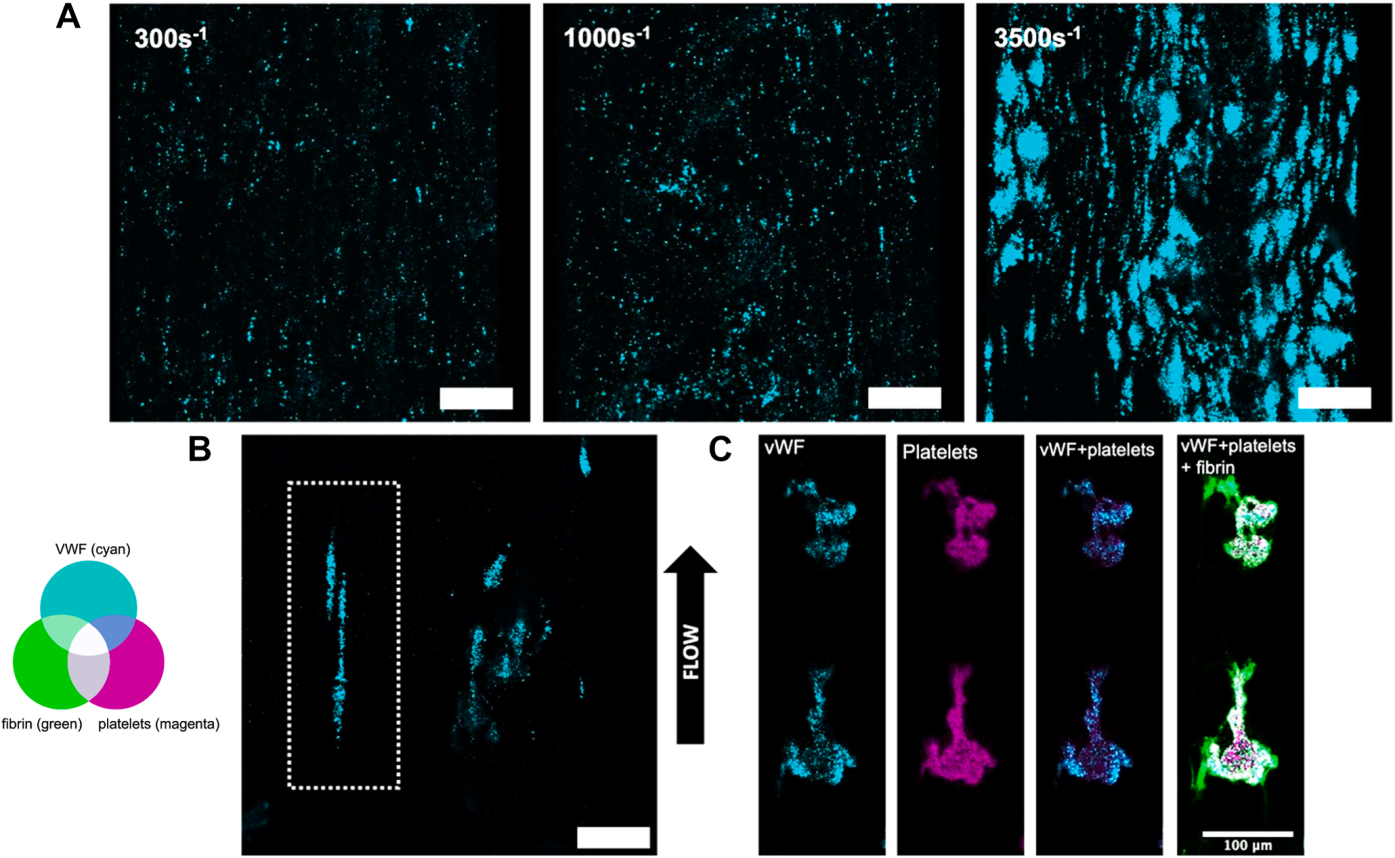
Impact of flow rate on von Willebrand factor (VWF) unfolding. (A) Images show VWF signal from clots formed under 300/s, 1000/s, and 3500/s at the end point of clot formation (after 10 minutes). The VWF intensity is the highest at the highest shear rate. Scale bar: 100 μm. (B) At the highest shear rate (3500/s), elongated VWF strands over the collagen surface were observed (marked by dotted lines) within the first minute of flow initiation. Both images show the first minute of flow, from 2 different experiments at 3500/s. Scale bar: 100 μm. (C) Fluorescent images of VWF (cyan), platelets (magenta), merged image of VWF and platelets, and a final merged image of VWF, platelets, and fibrin (green) at 3500/s after 10 minutes. VWF and platelets colocalize while fibrin surrounds the platelet aggregate. Scale bar: 100 μm.

The confocal data reveal a striking and rapid enhancement of platelet accumulation at 3500/s. A likely candidate mechanism is shear-sensitive unfolding of VWF [31]. To test this mechanism, we performed flow experiments from 1 of the donors, in the presence of a fluorescently tagged antibody specific for VWF. At the high (3500/s) shear rate, we observed clear colocalization of the platelet aggregates with VWF (Figure 3C). Elongated line–like VWF-associated features oriented in the direction of flow were visible, within a minute after initiating flow (Figure 3B, Supplementary Movie 2). By contrast, clots formed under lower shear rates (300/s or 1000/s) showed little VWF signal and no comparable flow-aligned linear features (Figure 3A). Together, these observations show that the enhanced platelet accumulation at 3500/s is accompanied by increased VWF-associated signal, consistent with greater VWF involvement under pathologic high-shear conditions.

### 3.3 Final clot composition and structure

**Figure 4:**
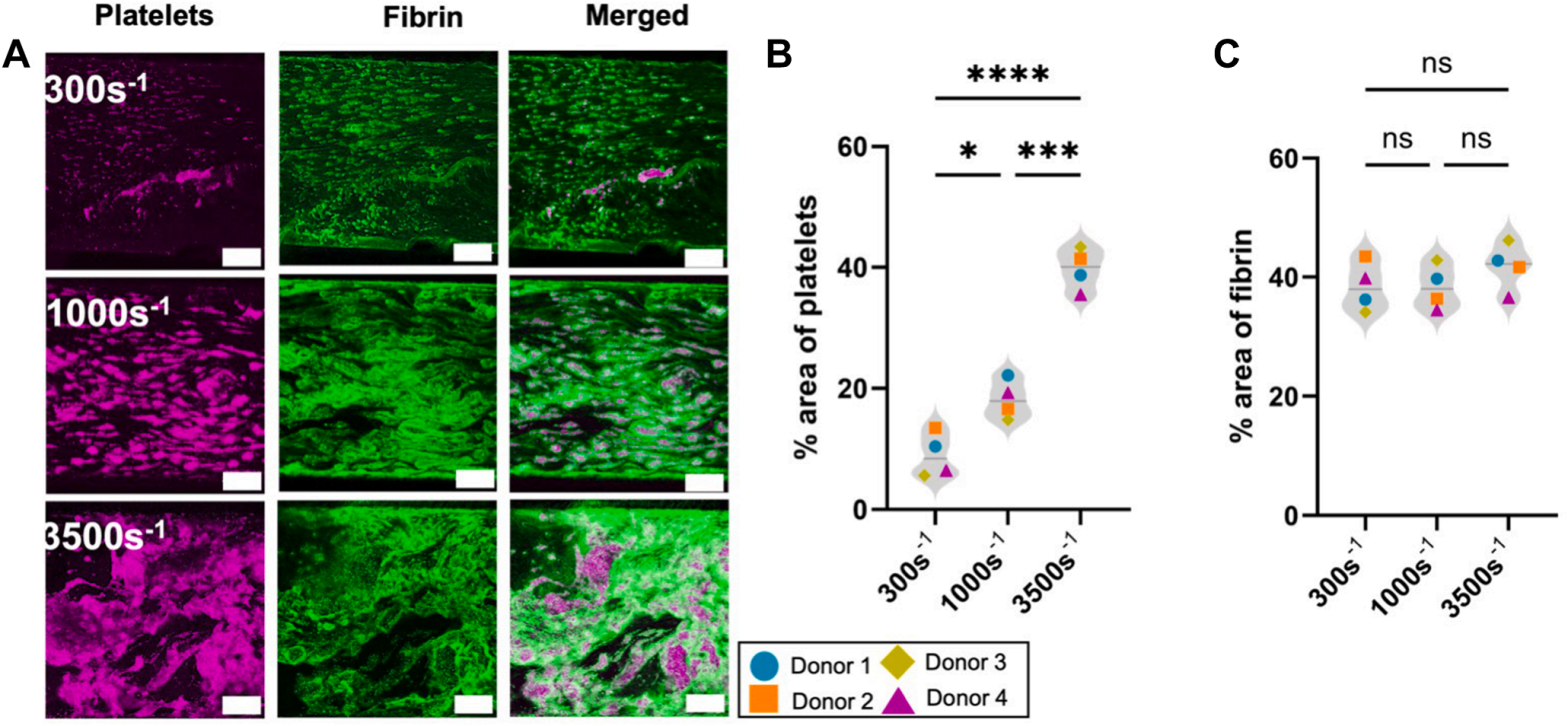
Final clot composition varies with shear rate. (A) Representative maximum intensity projections of clots formed under shear rates of 300/s, 1000/s, and 3500/s after 10 minutes of perfusion. Images display platelets (magenta) and fibrin (green). Scale bars: 100 μm. (B, C) Quantification of final clot composition in terms of the percentage of surface area covered by platelets (B) and fibrin (C) at each shear rate condition. Values for each flow rate are calculated by averaging minimum 4 replicate experiments from 4 individual donors. Higher shear rates yield clots that are significantly richer in platelets, while no clear trend is observed for fibrin coverage. ∗*P* < .05; ∗∗*P* < .005; ∗∗∗*P* < .0005; ∗∗∗∗*P* < .0001; ns, not significant.

To characterize the final composition of clots formed at different arterial shear conditions, we quantified the surface area covered by fibrin and platelets along with the size of platelet aggregates at the final (10 minutes) time point. Representative confocal images (Figure 4A) show that platelet deposition significantly increased as the shear rate was increased from 300/s to 3500/s. The platelet aggregates were sparse, small and spatially discrete at 300/s, more extensive with moderate-sized aggregates at 1000/s, and large and interconnected at 3500/s. Quantification of the final clot composition in terms of the percentage of surface area covered by platelets and fibrin at each shear rate condition showed that clots were significantly richer in platelets at the highest shear rate than in lower shear rates. Increasing shear led to a progressive increase in platelet surface coverage from 9.1% ± 1.4% at 300/s to 19.8% ± 1.1% at 1000/s and 39.2% ± 1.5% at 3500/s (Figure 4B).

Meanwhile, the fibrin signal showed no clear change with varying flow rates (Figure 4A). Quantification of the fibrin surface coverage confirmed that the shear rate did not influence fibrin deposition (Figure 4C). As a consequence, clots formed at the highest shear rate of 3500/s were relatively richer in platelets (ratio of fibrin-to-platelet coverage, 1.0), while clots formed at lower shear rates were richer in fibrin (ratio of fibrin-to-platelet surface coverage of 3.5 for 300/s and 1.6 for 1000/s). Quantification for each individual donor can be found in Supplementary Figure 4.

Comparing the fibrin and platelet signals, we clearly observed that the platelets were attached to the bottom surface and covered by fibrin (Supplementary Figure 5). This observation was consistent with the time-lapse images from Figure 2, which showed that platelets first adhere and aggregate on the coated surface, acting as nucleation sites for the subsequent formation of a fibrin network.

Additionally, shear rate influenced the height of the clots. This is illustrated in Figure 1A, which shows 3D reconstructions of the clots that are color coded for depth to visualize the clot height (additional 3D renderings in Supplementary Movie 3). The average height of the clots increased with increasing shear rate, from 29 ± 1 μm at 300/s to 40 ± 1 μm at 1000/s and 49 ± 1 μm at 3500/s (Figure 1B). This height increase implies an increased occlusive capacity: clots formed under 300/s reached 41% of the channel height, while at 1000/s, they reached 57% of the channel height, and finally, at 3500/s, they reached 70% of the channel height. Together with the increased clot height at increasing shear rate, the lateral platelet aggregate size increased significantly (Figure 1C). At 3500/s, the largest platelet aggregates had a mean size of 3120 ± 115 μm^2^. Moreover, the aggregates were interconnected to form larger networks. The average platelet aggregate sizes at 1000/s and 300/s shear rates were significantly smaller, measuring 578 ± 47 μm^2^ and 67 ± 6 μm^2^, respectively. Mean aggregate size was quantified from thresholded maximum intensity projections of the DiOC_6_ channel and therefore represents the lateral area of DiOC_6_-positive platelet aggregates. This measurement was used to compare platelet aggregate size between shear-rate conditions but does not exclude the likely possibility that other thrombus components colocalize with these aggregates.

Altogether, our data show that higher arterial shear rates enhance platelet recruitment and promote the formation of larger and taller platelet aggregates, thus enhancing the clot’s occlusive capacity.

### 3.4 Mechanical properties of clots

**Figure 5:**
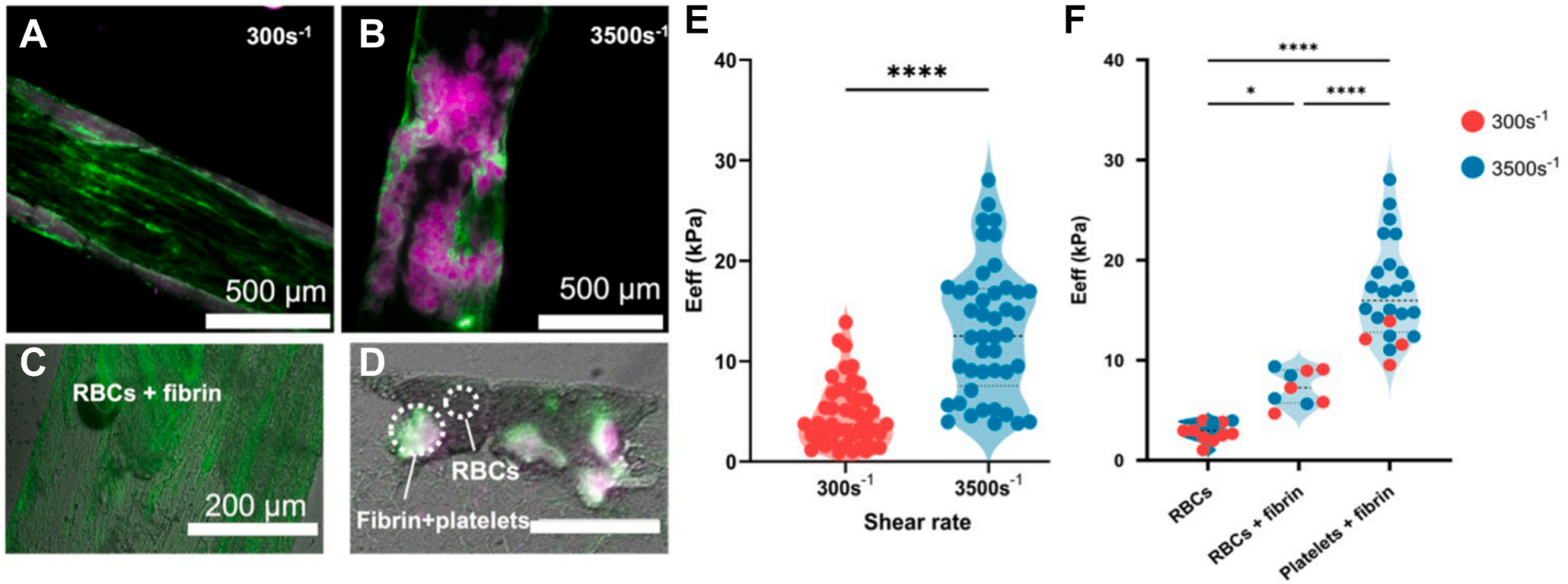
High shear rate clots are stiffer than low shear rate clots on average, but the clots show composition-dependent spatial variations in local stiffness in both cases. (A) Representative low (300/s) shear rate clot as imaged during indentation via a widefield fluorescent microscope (platelets, magenta; fibrin, green). Scale bar: 500 μm. (B) Corresponding example of a high (3500/s) shear rate clot. Scale bar: 500 μm. (C) Example of a local region rich in red blood cells (RBCs; brightfield) and fibrin within a low shear rate clot (green; fluorescent channel). Scale bar: 200 μm. (D) Example of a region rich in RBCs alone vs a neighboring region rich in both platelets and fibrin within a high shear rate clot (labeled with white text). RBCs shown in brightfield, fibrin in green, and platelets in magenta. Scale bar: 200 μm. (E) Effective Young modulus (*E*_eff_) of clots at low (300/s) vs high (3500/s) shear rates, averaged over 9 clots measured at 5 locations. (F) Effective Young modulus values pooled for the 2 shear rates, separated by local clot composition. Regions rich in fibrin and platelets are stiffer than regions that are poor in platelets but rich in RBCs (with or without fibrin). Regions that are rich in only RBCs are the softest. ∗*P* < .05; ∗∗∗∗*P* < .0001.

To determine the mechanical properties of the clots, we performed microindentation measurements on the clots using an indenter mounted on an inverted fluorescence microscope. We studied clots formed at the lowest (300/s) and the highest (3500/s) shear rates, for which confocal imaging revealed large compositional differences. Fluorescence imaging coupled to the indenter were consistent with the confocal images shown before, showing heterogenous clots at both shear rates and platelet-rich clots at higher shear rates. Simultaneous brightfield imaging revealed the presence of RBCs within the clot that did not always colocalize with fibrin or platelets, indicating the presence of regions rich in RBCs only. Quantification of the relative RBC surface coverage of these clots is presented in Supplementary Figure 6. At both shear rates, the clots were spatially heterogeneous, showing distinct areas that could be classified as RBC-rich (Figure 5D), RBC- and fibrin-rich (Figure 5C) or fibrin- and platelet-rich regions (Figure 5D).

To quantify the impact of the shear rate during clot formation on clot stiffness, we performed 5 indentations per clot and compared the average effective Young modulus (*E*_eff_). On average, clots formed under high shear (3500/s) had a significantly higher *E*_eff_ of 14.1 ± 1.4 kPa than those formed at low shear (300/s), with 4.1 ± 0.6 kPa (Figure 5E). However, since clots in both cases exhibited local compositional heterogeneity, we next evaluated the mechanical heterogeneity within the clots according to the 3 primary groups identified from the fluorescence and brightfield images (Figure 5C,D). As shown in Figure 5F, irrespective of shear rate, platelet- and fibrin-rich regions were significantly stiffer (17.5 ± 1.1 kPa) than areas dominated by fibrin and RBCs (6.8 ± 0.6 kPa). Regions rich in RBCs alone showed the lowest average *E*_eff_ of 3.1 ± 0.3 kPa, underscoring the key mechanical contribution of platelet aggregates. In conclusion, the higher average stiffness of clots formed under high shear (3500/s) compared with those formed at low shear (300/s) can be explained by the prevalence of areas rich in large platelet aggregates formed under higher flow.

## 4 Discussion

In this study, we developed a microfluidic arterial thrombosis model to investigate how varying arterial shear rates influence clot composition, clot formation dynamics, and local mechanical properties. By combining real-time fluorescent imaging with postformation microindentation, we provide an integrated view of thrombus development and structure along with mechanical heterogeneity under physiologically and pathologically relevant flow conditions.

### 4.1 Mechanism of clot formation and final clot characteristics are shear dependent

**Figure 6:**
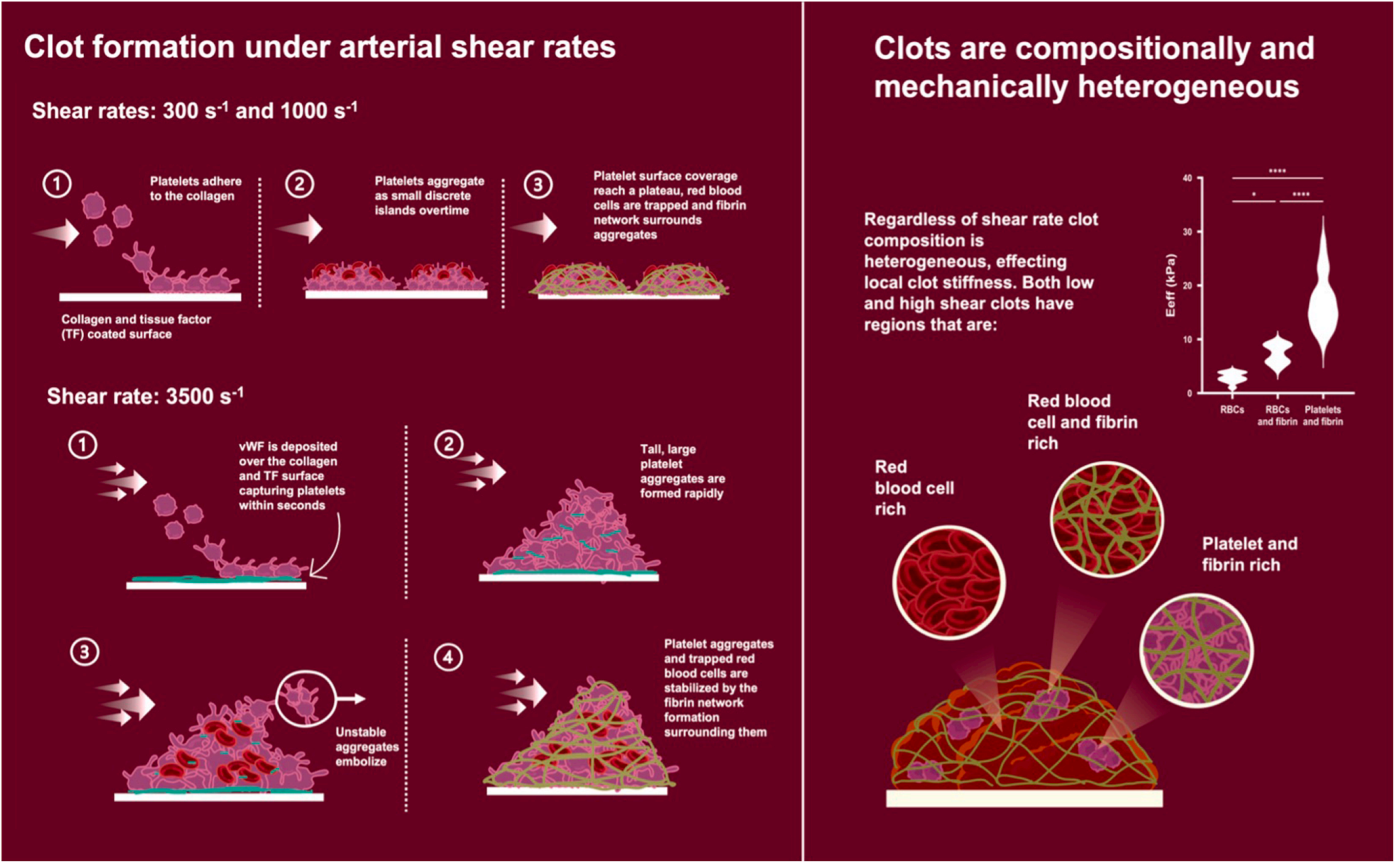
Summary of the clot formation mechanism observed under physiological (300/s and 1000/s) and pathologic (3500/s) shear rates. At 300/s and 1000/s the clot formation: (1) initiates by platelet adhesion over the coated surface, (2) followed by aggregation of platelets into islands that begin entrapping red blood cells; the aggregate size increases with increasing shear rate and reaches a plateau when (3) fibrin network forms around the aggregates. At 3500/s, where the shear rate is high enough for unfurling VWF: (1) VWF strands are deposited over the coated surface facilitating platelet capture and adhesion, (2) tall, large platelet aggregates rapidly form, (3) unstable platelet aggregates embolize under high shear forces, and (4) finally, they are stabilized by the fibrin network. Regardless of the shear rate, clot composition shows local heterogeneity, affecting its local stiffness. On average, high shear rate clots are significantly stiffer than low shear rate ones. Both high and low shear rate clots have regions that are red blood cell rich, red blood cell and fibrin rich, and platelet and fibrin rich, which show the lowest and the highest local stiffness responses, respectively.

Time-lapse imaging of the clotting process showed a clear sequence of events, as summarized in Figure 6. At physiological shear rates (300/s and 1000/s), clotting followed 3 main steps: (1) platelet adhesion on the coated surface, (2) platelet aggregation into discrete islands that expand across the surface, and (3) fibrin network formation that surrounds and interconnects aggregates, reinforcing the structure. At 300/s, overall platelet coverage remained low across 10 minutes and fibrin formed around relatively small aggregates. At 1000/s, platelet coverage increased and reached a plateau with fibrin network formation. RBC retention followed the initial platelet aggregation, as the RBCs colocolized with platelet aggregates. Fibrin network formation then stabilized both the platelets and the entrapped RBCs, while streams of flowing RBCs could still move in between these aggregates, represented in Supplementary Movies 4 to 6. Our observation that fibrin surface area coverage did not differ among 300/s, 1000/s, and 3500/s appears to contrast with previous reports showing shear-dependent effects on fibrin formation and architecture. However, these differences are likely explained by important methodological differences between studies. Neeves et al. [28] used a purified fibrinogen/thrombin flow system, without platelets, RBCs, plasma coagulation factors, or a TF-bearing surface and showed that increasing shear rates from (10/s to 100/s) reduced fibrin deposition and fiber thickness between 10/s and 100/s shear rates [28]. In that system, fibrin formation is strongly governed by transport of thrombin and fibrin monomers, whereas in our model, local thrombin generation from the collagen–TF surface and the presence of platelet aggregates as anchors are likely sufficient to overcome shear-dependent washout across the tested range.

Other studies investigating thrombus formation under flow have often used native or differently anticoagulated whole blood, different thrombogenic surfaces, and readouts such as fibrin deposition, thrombus volume, fiber thickness, alignment, or ultrastructure rather than 2-dimensional fibrin surface area coverage [17,28,32]. For example, Chernysh et al. [17] studied thrombi from arterial vs venous *ex vivo* thrombi, showing that flow conditions can alter fibrin organization, promoting denser or more aligned fibrin structures, particularly in platelet-rich arterial thrombi [17]. Both Neeves et al. [28] and Chernysh et al. [17] used higher-magnification imaging to investigate the fibrin network properties. Such changes are not detected by our end point measurement, since surface area coverage reports whether fibrin is present on the surface but does not capture fibrin fiber thickness, network density, clot height, or 3D architecture. Therefore, our findings suggest that under collagen–TF-driven coagulation in recalcified citrated whole blood, fibrin surface coverage is not strongly shear-dependent, but they do not exclude shear-dependent changes in fibrin network structure or thrombus composition. Future studies using higher-resolution imaging, such as electron microscopy, may help determine whether shear affects fibrin architecture, density, fiber thickness, and organization despite the absence of changes in overall surface area coverage.

At pathologically high shear rates (3500/s), these same steps occurred, but the initiation and growth phases were accelerated and driven by VWF. Based on previous studies of the mechanosensitivity of VWF [31,33], 3500/s is above the shear rate threshold for VWF unfurling. At 3500/s, already within the first minute of flow, we observed VWF strands in the direction of flow, deposited over the coated surface. These strands were absent at lower rates. VWF is known to facilitate platelet capture over thrombogenic surfaces, such as collagen, at high shear rates [34,35]. We observed these strands mainly at the bottom of the coated channel surface, indicating that they form an initial bridge between collagen and platelets. This is further strengthened by the observation that the platelets and VWF colocalize in these clots. Additionally, clots formed at 3500/s exhibited a pronounced increase in platelet aggregation and VWF signal compared with clots formed at the 2 lower shear rates. This can be explained by the ability of VWF to facilitate platelet–platelet aggregation at higher shear rates by tethering circulating platelets to the already adhered platelets [14,34]. Following the rapid platelet aggregation step, an additional step emerged at this high shear: a transient unstable/embolization phase. During this unstable phase, adhered platelets detached and were carried away by the flow, causing reorganization of the platelet aggregates. While not all 3500/s clots showed this behavior, 6 of 16 clots did, whereas no similar behavior was observed in lower shear clots. This phase likely reflects the mechanical challenge faced by rapidly forming, tall platelet aggregates exposed to high shear stresses. Clots formed at 300/s and 1000/s are shorter and composed of smaller platelet aggregates, consistent with the results of the study by Pero et al. [19], who reported increased platelet surface coverage and clot height when shear was raised from 500/s to 1000/s. The larger clot height at 3500/s likely amplifies local shear rate, reinforcing platelet recruitment, whereas the shorter structures at lower shear experience less disruptive stress. This observation aligns with classical shear-induced platelet aggregate mechanisms described by Nesbitt et al. [13], who observed cycles of platelet aggregation and disaggregation in an *in vivo* mouse stenosis model [13]. Similarly, Colace et al. [36] showed a flow model where they first formed an initial stable platelet layer over a collagen surface at 200/s and then increased the flow rate to >7800/s, triggering a rapid (within 100 seconds) and unstable platelet aggregation and embolization with a simultaneous increase in the VWF signal. In the same system, inhibition of GPIbα or α_IIb_β_3_ abolished this unstable rapid aggregation and embolization burst. In our system, this unstable phase resolved when fibrin network matured (∼6 minutes) and stabilized platelet coverage. Our data strongly suggest that the formation of a fibrin scaffold plays a crucial role in stabilizing aggregates and halting further dynamic remodeling of the clot. These findings are consistent with prior work demonstrating that fibrin crosslinks and consolidates platelets, resisting disintegration and removal by flow [16,37,38].

### 4.2 Clot composition determines local stiffness, with platelet-rich regions being stiffer

Indentation measurements showed that clots formed under high shear (3500/s) were on average 3.4-fold stiffer than clots formed at low shear (300/s). This finding is consistent with an earlier study, where clots formed in a stenotic microfluidic model were 2.4-fold stiffer when formed under high shear (3500/s) than those formed at low shear flow [21]. We observed marked regional mechanical heterogeneity for clots across all shear conditions. This mechanical heterogeneity covaried with local clot composition: regions that were rich in both platelets and fibrin were significantly stiffer than regions dominated by fibrin and RBCs, while regions mainly rich in RBCs were softest. Similar heterogeneity was observed in thrombi retrieved from patients [17,39]. We therefore attribute the larger stiffness of clots formed at higher shear to their larger platelet content and platelet-to-fibrin ratio, clearly observed by imaging. Although we did not measure contraction directly, platelet-driven clot contraction is known to compact fibrin and RBCs, leading to polyhedrocyte formation, providing a plausible mechanistic link between platelet content and increased stiffness [[40], [41], [42]]. Higher content of platelets in high shear rate clots could have lead to increased stiffness due to increase in overall contractile forces within the clot. Indentation measurements by Swieringa et al. [16] on clots formed in a microfluidic device with a single channel similarly revealed stiffer clots at high (1000/s) when compared with those at low (150/s) shear with a larger platelet-to-fibrin ratio. Our observation of lower Young moduli for RBC-rich regions aligns with previous macroscopic compression and tensile measurements by Cahalane et al. [20] for static clot analogs with varying RBC content, where increasing RBC concentration lowered the clot stiffness. Our observations are likewise consistent with analyses of human ischemic stroke thrombi, which reported a strong positive correlation between fibrin/platelet content and stiffness and an inverse correlation between RBC content and stiffness [4]. Altogether, our findings confirm the key contribution of platelet aggregates to thrombus stiffness, as previously observed in *ex vivo* thrombi and *in vitro* static clots [6,7,20]. Future work should more directly assess clot constituents beyond platelets and fibrin, including immune cells, neutrophil extracellular traps (NETs), and phosphatidylserine-positive platelets. Prior work on retrieved stroke thrombi indicates that leukocytes and extracellular DNA-/NET-associated material are enriched predominantly in platelet-rich regions and at platelet-rich/RBC-rich interfaces, while RBC-rich regions are structurally simpler and contain fewer leukocytes and little extracellular DNA [22,43]. Moreover, NET enrichment has been associated with increased stiffness in stroke clot analogs [44]. These observations raise the possibility that such components may contribute to the elevated local stiffness observed in platelet-rich clot regions, and direct histologic or immunophenotypic analysis across shear conditions will therefore be valuable in future studies. An important next step in this work will be to quantify clot lysis time during perfusion of exogenous tissue-type plasminogen activator over preformed clots. Flow-based and microfluidic studies have shown that real-time assessment of thrombolysis under controlled perfusion can provide important insight into clot dissolution kinetics [45,46] and that fibrinolysis under flow may exhibit spatially heterogeneous and evolving lysis behavior [47,48].

Traditional analyses of patient-derived clots are mainly performed on thrombi that are formed after embolization, altered due to thrombectomy procedure or treatment. Furthermore, it is not possible to identify the structural differences due to the time delay between analysis and thrombus formation. In this study, we demonstrated an experimental platform to structurally generate and to mechanically study blood clots from the moment of formation under flow, offering a more complete and unbiased perspective of thrombus evolution. Furthermore, microindentation allows spatially resolved mechanical mapping, which is not achievable through bulk testing. This approach may offer new insight into which regions of a thrombus are most vulnerable to embolization or most resistant to thrombectomy.

### 4.3 Limitations

While this flow model replicates arterial shear conditions and offers high-resolution imaging, there are several aspects where it differs from the *in vivo* situation: it does not include an endothelial cell layer nor pulsatile or gradient flow, which can modulate platelet adhesion and fibrin formation *in vivo* [49,50]. Because our straight-channel model imposes defined shear rates rather than stenosis-like shear gradients, it does not capture the additional prothrombotic effects of shear-rate gradients, which have been shown to accelerate VWF-dependent platelet aggregation and thrombus formation [50]. Hematological parameters, including platelet count and hematocrit, were not recorded for individual donors, and we cannot rule out that donor-to-donor variation in these values may have contributed to variability in thrombus formation. All experiments were conducted at room temperature, which may underestimate the amount of platelet adhesion, aggregation, and contraction [51]. Similarly, the rectangular cross-section of the channel does not fully recapture the *in vivo* circular vessels, leading to a nonuniform distribution of wall shear stress toward the corners of the channel [52]. The microindentation analysis assumes Hertzian contact with a homogeneous, purely elastic material; given the viscoelastic and porous nature of clots [53,54], the reported *E*_eff_ values should be interpreted in relative rather than absolute terms. RBC-rich regions were classified using brightfield imaging only, limiting RBC specifity and quantification; more quantitative staining could refine the assessment of RBC content varying with shear rate. Although DiOC_6_ signal in our flow assay was visually dominated by platelets, its lack of platelet specificity means that rare leukocyte labeling cannot be completely excluded, such events were infrequent and unlikely to meaningfully affect surface area measurements. Another minor limitation is that potential DiOC_6_-associated phototoxic platelet activation was not directly assessed under our imaging conditions; however, all experimental groups were labeled and imaged using the same protocol.

## 5 Conclusion

We studied clot formation across a range of arterial shear rates, revealing how, within the arterial regime, shear rate influences clot structure, growth dynamics, and mechanical properties. Our findings reveal that increasing shear rates not only promote faster formation of larger platelet aggregates but also introduce a transient instability phase prior to fibrin stabilization at pathologically high shear rates. We further demonstrate that platelet- and fibrin-rich regions are significantly stiffer than RBC-dominant regions, underscoring how compositional heterogeneity translates into mechanical heterogeneity at the microscale. By enabling observation and analysis of the full course of clot development under physiologically relevant conditions, our platform bridges an important gap by allowing local mechanical characterization coupled with compositional information to account for heterogeneity of thrombi.
